# Successful rescue of fulminant myocarditis with mechanical circulatory support and immunosuppression therapy: A case report

**DOI:** 10.3389/fcvm.2023.1144630

**Published:** 2023-04-19

**Authors:** Chi Zhou

**Affiliations:** Division of Cardiology, Department of Internal Medicine, Tongji Hospital, Tongji Medical College of Huazhong University of Science and Technology, Wuhan, China

**Keywords:** fulminant myocarditis, mechanical circulatory support, immunosuppression, case report, cardiogenic shock

## Abstract

Myocarditis is challenging to diagnose because of its various clinical manifestations. Fulminant myocarditis (FM) is a severe type of myocarditis characterized by heart failure, malignant arrhythmia, cardiogenic shock, and cardiac arrest. Its early diagnosis and timely treatment are crucial for a positive long-term prognosis. Here we report a case of 42-year-old woman who presented with fever, chest pain, and cardiogenic shock. An initial examination showed increased myocardial enzyme levels and diffuse ST-segment elevation. Urgent coronary angiography excluded coronary artery stenosis. Echocardiography revealed decreased left ventricular systolic function. Cardiac magnetic resonance imaging revealed cardiomyocyte necrosis and interstitial inflammatory edema. The patient was diagnosed with FM and administered antiviral and anti-infective agents, glucocorticoid, immunoglobulin, and supported with temporary cardiac pacemaker and positive airway therapy, and treated with continuous renal replacement therapy. As her clinical condition deteriorated rapidly, we immediately started an intra-aortic balloon pump and veno-arterial extracorporeal membrane oxygenation. She was discharged on day 15 and recovered normally during follow-up. The early initiation of mechanical circulatory support and immunosuppression are life-saving tools for the treatment of FM.

## Introduction

Myocarditis is a myocardial inflammatory injury caused by infections, autoimmune diseases, and poisoning (1). The global prevalence of myocarditis is 8–10 cases per 100,000 individuals annually (2). A study, describing autopsy findings of young people who suffered sudden cardiac death, reported a high proportion of myocarditis. However, myocarditis is a challenging diagnosis because it exhibits various clinical symptoms including fever, weakness, chest pain, palpitation, dyspnea, and diarrhea (3). Viral infection is the most common cause of myocarditis, and the coxsackie virus B, adenovirus, cytomegalovirus, Epstein-Barr virus, and influenza virus are the predominant pathogens (4).

Fulminant myocarditis (FM) is a severe type of myocarditis with rapid disease progression resulting in hemodynamic disorders, circulatory failure, malignant arrhythmia, cardiogenic shock, and cardiac arrest (5). The FM heart is characterized by a thickened and non-dilated ventricle with systolic dysfunction due to cardiomyocyte necrosis, intense inflammation, and interstitial edema. FM patients show high early mortality but excellent long-term prognosis (6). Therefore, early diagnosis and timely treatment are important for its management. Coronary angiography (CAG), cardiac magnetic resonance (CMR) and endomyocardial biopsy (EMB) are frequently used to diagnose FM. The major therapeutic strategies for FM currently include guideline-directed medical therapy for heart failure, anti-arrhythmic medications, and specific therapies directed to particular etiology. Our clinical experience indicated that FM should be treated with comprehensive strategies, including immunosuppression by glucocorticoid and immunoglobulin, pacemaker and respirator support, continuous renal replacement therapy (CRRT), and mechanical circulatory support (intra-aortic balloon pump [IABP] and veno-arterial extracorporeal membrane oxygenation [VA-ECMO]) (1, 2, 4, 7).

Here, we report a case of cardiogenic shock in a young female FM patien that was successfully rescued with mechanical circulatory support (IABP + VA-ECMO) and immunosuppression therapy.

## Case presentation

**Figure 1:**
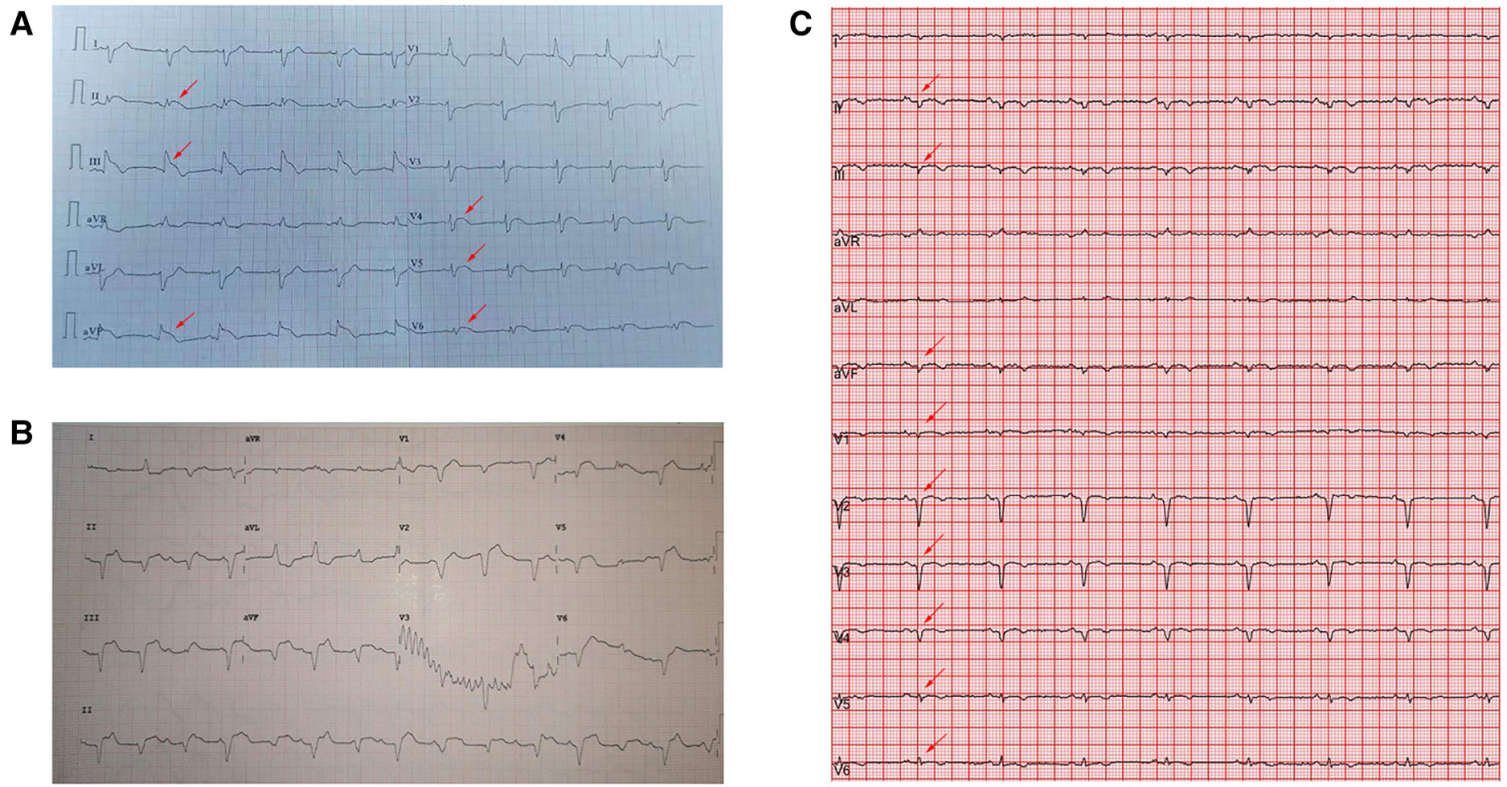
Electrocardiography showed (**A**) ST-T elevation (red arrow) in the anterolateral and inferior leads in the municipal hospital. (**B**) Type III atrioventricular block on admission. (**C**) Sinus rhythm with Q waves (red arrow) in the anterior and inferior leads on day 7 of hospitalization.

**Figure 2:**
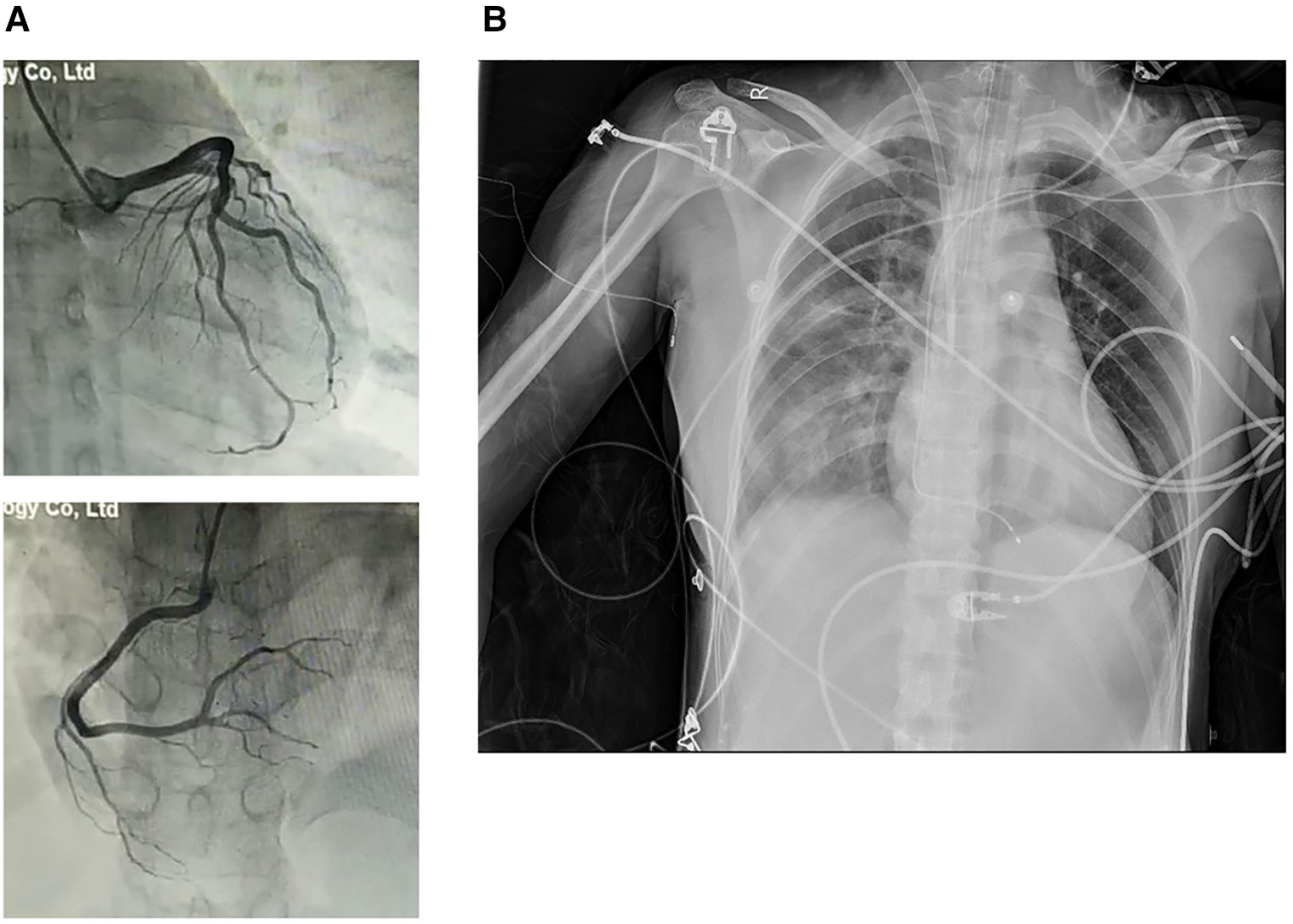
(**A**) CAG excluded coronary artery stenosis. (**B**) Chest radiography revealed enlarged cardiac size and a lung infection.

A 42-year-old previously healthy woman was admitted to Wuhan Tongji Hospital in July 2018 with a 3-day history of fever and chest pain. Her peak temperature was 39.0°C. She did not experience cough, nausea, vomiting, syncope or diarrhea. She had been treated with piperacillin and acetaminophen at a nearby clinic. Nevertheless, her condition deteriorated within 2 days, and she was immediately sent to a local hospital. Electrocardiography showed ST-T elevation in the anterolateral and inferior leads at that time (Figure 1A). The cardiac troponin I (cTnI) level was >50,000 pg/ml. She was suspected to have acute myocardial infarction, but urgent CAG excluded coronary artery stenosis (Figure 2A). The patient was transferred to our hospital for further treatment at 3 days after the initial discomfort. She had no history of hypertension, diabetes, pneumonia, gastrosis, bleeding disorders, kidney disease, trauma, toxin exposure, or allergies before onset.

On arrival, her vital signs were as follows: body temperature 36.0°C, blood pressure (BP) 79/50 mmHg, pulse 125 beats/min, respiratory rate 25 breaths/min, and oxygen saturation (SPO_2_) 92% (room air). A physical examination revealed engorged neck veins. No cyanosis was noted. Respiratory movements were bilaterally symmetrical without wheezing or rales. The heart sounds were weak and the cardiac rhythm was regular. No pathological murmurs were observed. The abdomen was flat and soft without pain. No liver and spleen enlargement was observed. There was no pitting edema and pathological signs were negative. Admission laboratory tests showed: cTnI >50,000 pg/ml, myoglobin 328.7 ng/ml, creatine kinase-MB 243.1 ng/ml, and *N*-terminal pro-brain natriuretic peptide (NT pro-BNP) 8,094 pg/ml. Blood routine examination showed leukocyte count 12.79 × 10^9^/L, neutrophil count 10.69 × 10^9^/L, lymphocyte count 0.98 × 10^9^/L, and eosinophil count 0.02 × 10^9^/L. Other laboratory test findings were as follows: alanine transaminase (ALT) 40 U/L, aspartate transaminase (AST) 359 U/L, creatinine 53 µmol/L, lactic dehydrogenase 889 U/L, D-dimer 1.10 µg/ml, lactic acid 5.34 mmol/L, and C-reactive protein 118.8 mg/L. The patient was in Society for Cardiovascular Angiography and Interventions (SCAI) shock C stage. Electrocardiography revealed a type III atrioventricular block (Figure 1B). Transthoracic echocardiography showed diffuse ventricular hypokinesia [left ventricular ejection fraction (LVEF) 26%] and pericardial effusion (Supplementary Video S1A). Chest radiography revealed enlarged cardiac silhouette and pulmonary infiltrates (Figure 2B). In addition, etiologic tests for coxsackie virus B, adenovirus, cytomegalovirus, Epstein-Barr virus, influenza virus, hepatitis, HIV, and syphilis were negative. We did not perform an pulmonary artery catheter because consent was not provided by the patient.

Based on these clinical findings, the patient was suspected to have FM and admitted to the cardiac care unit. A temporary cardiac pacemaker was immediately implanted to correct the atrioventricular block. Intravenous dopamine (10 μg/kg/min) was administered in response to the hypotension. Unfortunately, the vasopressor failed to maintain her BP (90/52 mmHg). An emergency IABP machine connected to 34 cc balloon was inserted *via* the right femoral artery, with a counterpulsation ratio of 1:2. Her symptoms gradually resolved, with a counterpulsation BP of 108 mmHg. High-dose methylprednisolone (200 mg/day) and intravenous immunoglobulin (20 g/day) were administered for immunosuppression. Penciclovir (0.25 g q12h), oseltamivir (75 mg bid), and biapenem (0.3 g q8h) were chosen as antiviral and anti-infective treatments. Other drugs included trimetazidine, coenzyme Q, vitamin C, pantoprazole, and furosemide.

On the second morning, the patient's fever returned at 39.5°C. The counterpulsation pressure decreased to 54 mmHg, SPO_2_ 80% with audible moist rales. Telemetry monitoring revealed short ventricular tachycardia. An arterial blood gas analysis revealed type I respiratory failure (pH 7.396, partial pressure of CO_2_ [PaCO_2_] 35.1 mmHg, partial pressure of O_2_ [PaO_2_] 54.9 mmHg, HCO_3_ 21.5 mmol/L and blood base excess [BE] −2.7 mmol/L). Laboratory test results worsen: cTnI >50,000 pg/ml, NT pro-BNP 28,282 pg/ml, ALT 668 U/L, AST 1,257 U/L, creatinine 116 µmol/L, lactic dehydrogenase 1,591 U/L, and lactic acid 7.90 mmol/L. The patient presented with dyspnea, dysphoria and anuria. The patient was in SCAI shock D stage. We administered a noninvasive respirator (continuous positive airway pressure 12 cm H_2_O) to provide adequate tissue oxygenation and increased the dosage of dopamine (20 μg/kg/min). Echocardiography showed severe LV systolic dysfunction. After obtaining informed consent, we initiated V-A ECMO *via* the left femoral artery and vein. The initial settings were: 3,500 rpm, 45% and 5 L/min of oxygen. Heparin was continuously administered to maintain an activated partial thromboplastin time of 70–100 s. An intermittent transfusion was performed to ensure that the hemoglobin level was >90 g/L. CRRT with a specialized hemofilter (AN69ST, oXiris, Baxter, France) was used to optimize intravascular volume and filter inflammatory mediators. The system includes a highly cytokine and endotoxin adsorptive membrane.

**Figure 3:**
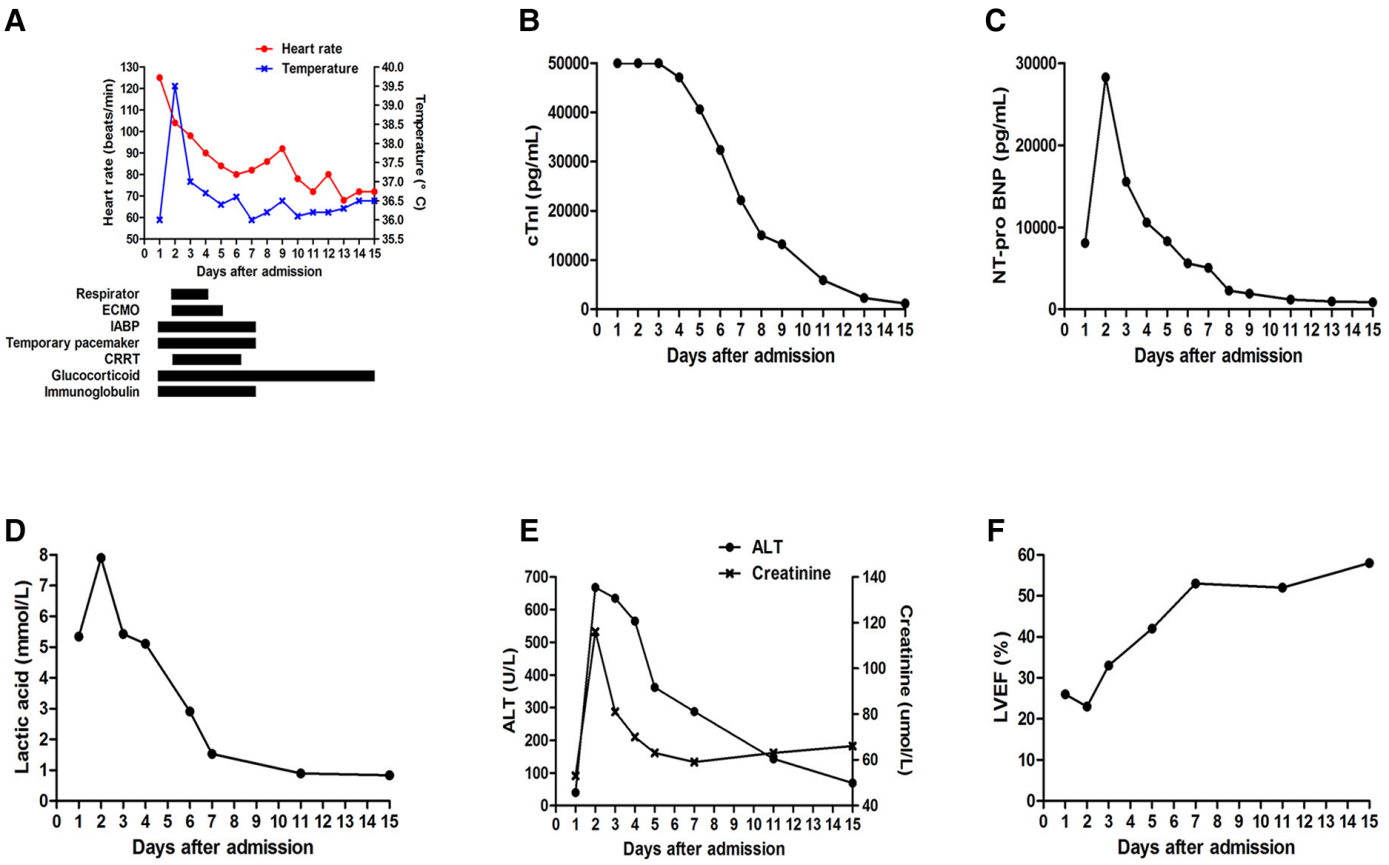
(**A**) Combined treatment of IABP, VA-ECMO, temporary pacemaker, respirator, CRRT with immunosuppression therapy. Changes in (**B**) cTnI, (**C**) NT pro-BNP, (**D**) lactic acid, (**E**) ALT and creatinine, (**F**) LVEF during hospitalization.

**Figure 4:**
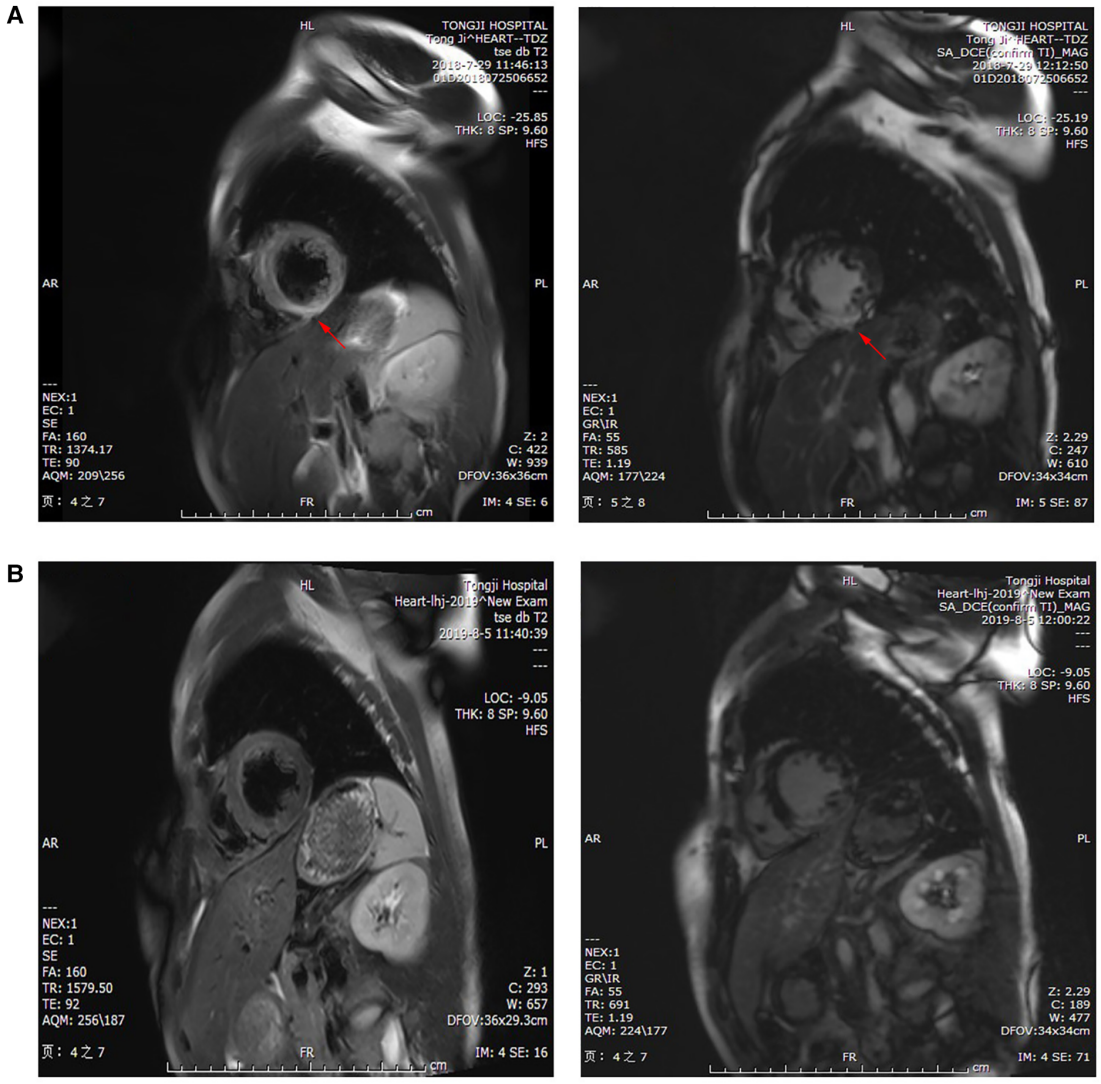
CMR examination showed (**A**) cardiomyocyte necrosis, diffuse edema, ventricular wall thinning (red arrow), and reduced movement in the left ventricle on day 10 of hospitalization, and (**B**) slight ventricular septal fibrosis and inflammatory edema at 1 year.

The subsequent days of treatment used a combination of IABP, VA-ECMO, temporary pacemaker, respirator, and CRRT with immunosuppression therapy (Figure 3A). The patient's body temperature was normal, her urinary output increased, and her water balance tended to be negative. The cardiac injury marker cTnI (Figure 3B), NT pro-BNP (Figure 3C), lactic acid (Figure 3D), ALT, and creatinine (Figure 3E) gradually decreased. Echocardiography indicated a progressively improved LVEF (Figure 3F). The dopamine dosage and VA-ECMO rate were gradually tapered according to BP. We reduced methylprednisolone 120 mg/day and immunoglobulin 10 g/day on day 4, reduced methylprednisolone 40 mg/day and stopped the immunoglobulin on day 7, and then switched to oral prednisone on day 9. Antiviral and anti-infective drugs were administered for 10 days. On day 4, the arterial blood oxygen increased (pH 7.414, PaCO_2_ 37.6 mmHg, PaO_2_ 82.5 mmHg, HCO_3_ 23.6 mmol/L and BE −0.1 mmol/L), and the respirator was pulled out. Considering that the BP could be maintained at 1,500 rpm on day 5, the VA-ECMO support was stopped. On day 7, laboratory tests improved as follows: cTnI 22,194 pg/ml, NT pro-BNP 5,067 pg/ml, ALT 288 U/L, AST 84 U/L, creatinine 59 µmol/L, lactic dehydrogenase 878 U/L, and lactic acid 1.53 mmol/L. Echocardiography showed improved ventricular wall motion (LVEF 53%), while electrocardiography showed sinus rhythm with Q waves in the anterior and inferior leads (Figure 1C). The IABP and temporary pacemakers were discontinued. A CMR examination performed on day 10 revealed cardiomyocyte necrosis, diffuse edema, ventricular wall thinning, and decreased contractility of left ventricle (Figure 4A). We did not perform an EMB because consent was not provided by the patient.

The patient was discharged from the hospital on day 15 without apparent complications. Before discharge, her body temperature was normal and she reported no chest pain or dyspnea. Echocardiography indicated a normal cardiac chamber size and ventricular motion, with a LVEF of 58% (Supplementary Video S1B). Laboratory tests showed the following: cTnI 1,189.6 pg/ml, NT pro-BNP 872 pg/ml, ALT 69 U/L, AST 40 U/L, and creatinine 66 µmol/L. The following oral drugs were prescribed at discharge: prednisone, trimetazidine, coenzyme Q, metoprolol, and perindopril. The patient completed progressive, active physical rehabilitation at home.

During the 1-year follow-up period, no deterioration in cardiac function, recurrent myocarditis, heart failure, or fatal ventricular arrhythmias were noted. Laboratory tests showed values of cTnI 311.8 pg/ml at 1 month, 38.6 pg/ml at 3 month, and 22.6 pg/ml at 6 month. NT pro-BNP levels were 517 pg/ml at 1 month and 189 pg/ml at 6 month. We observed normal ventricular function and chamber size after 1 year. Nevertheless, a CMR examination revealed slight ventricular septal fibrosis and inflammatory edema at 1 year (Figure 4B).

## Discussion

FM is a life-threatening disease with high mortality rate. Here we described the successful rescue of a FM case that manifested as fever, chest pain, atrioventricular block, and cardiogenic shock, which was treated with mechanical circulatory support and immunosuppression. This strategy combines IABP and VA-ECMO with glucocorticoid and immunoglobulin.

The pathophysiological process of myocarditis is divided into acute, subacute and chronic phases. In the acute phase, which lasts 3–5 days, viral invasion and replication cause severe damage to the myocardium. The subacute phase is characterized by an immunological reaction. A few patients enter the chronic phase and present with chronic inflammation, contractive fatigue, myocardial fibrosis, and cardiomegaly (7, 8). The precursor symptoms of myocarditis, such as fever, cough, and dyspnea, are usually similar to those of the common cold. FM accounts for approximately 10% of all myocarditis cases, which rapidly deteriorates to heart failure, malignant arrhythmia, and cardiogenic shock within several days (9). In this case, the patient experienced hemodynamic instability 3 days after the fever and chest pain. Therofore, the early diagnosis and comprehensive treatment of FM are crucial.

Although EMB is the golden standard for the diagnosis of myocarditis, it is not recommended during the hemodynamic instability or life-threatening stage (1). EMB could help to verify specific myocarditis types and detect viral genomes. As we failed to conduct an EMB and the etiologic tests for the most common virus were negative, the final etiology of this patient was not confirmed. Based on the epidemiology and clinical manifestations, we suspected viral infection. CAG was performed emergently to rule out acute myocardial infarction. An non-invasive CMR examination is useful for diagnostic and prognostic considerations in patients with suspected FM. CMR-specific findings include myocardial edema, hyperemia or global relative enhancement as well as myocardial fibrosis or late gadolinium enhancement (10). Electrocardiography has high sensitivity but low specificity for identifying FM, which usually manifests as ST-T segment elevation, ventricular tachycardia, and atrioventricular block. Transthoracic echocardiography helps rule out non-inflammatory heart diseases. FM appears as non-dilated, thickened, and contractive fatigue in the left ventricle (11). In addition, troponin, myoglobin, creatine kinase-MB, NT pro-BNP, lactic acid, and cytokines often increase in FM. However, there is no obvious enzyme peak in FM patients as it is a progressive change. The pulmonary artery catheter is an important diagnostic and management tool for the FM-induce cardiogenic shock. But it is regrettable that we failed to perform because the patient refused. The diagnostic criteria for FM require a combination of clinical manifestations and laboratory and imaging findings.

The antiviral therapy in FM patients is controversial and should be performed according to clinical experience. Myocarditis is a myocardial inflammatory injury caused by infections, autoimmune diseases, and poisoning, while viral infection is the most common aetiology. A multicentre study revealed that viral genome was detected in 38% of EMB samples from myocarditis patients (12). In this case, the patient had no history of toxin exposure or allergies bofore this onset. As negative serological results cannot exclude possible viral infections, we suspected viral infection and recommended antiviral treatment. High-dose intravenous immunoglobulin (10–20 g/day) is recommended to regulate the immune response and rescue the dying myocardium (13). These findings were supported by the antiviral and anti-inflammatory effects of the immunoglobulin light chain in mice with viral myocarditis (14). The use of glucocorticoid is controversial, as it may facilitate viral replication. However, glucocorticoid can stimulate interferon secretion, inhibit immune reactions, prevent shock, protect multiple organs from injury, and alleviate cardiomyocyte necrosis (15). It is suggested to start with 200 mg/day methylprednisolone for 3–5 days, and then gradually reduce the dosage. The combination of immunoglobulin and glucocorticoid helps improve circulatory dysfunction and reduce cytokine storm and mortality in FM patients (16).

Life-support therapy included circulatory support, respiratory support, and renal replacement. Mechanical circulatory support is the core component of comprehensive therapeutic strategies for FM. IABP should be implanted as soon as possible in FM patients with unstable hemodynamics, as it can increase coronary perfusion and brain oxygen supply, increase BP, and reduce cardiac afterload, thus decreasing the need for vasoactive drugs in the acute phase (17). VA-ECMO should be considered timely to ensure rapid and comprehensive cardiopulmonary assistance if the IABP cannot sufficiently improve the cycle (18). The combination of VA-ECMO and IABP promotes cardiac function recovery and increases the survival rate (19). In this case, IABP + VA-ECMO was quickly initiated when the circulation remained inadequate after optimal medical therapy. The atrioventricular block occurred within the day before admission. Patients with bradycardia and atrioventricular block should be administered temporary pacemaker (7). The temporary pacemaker was utilized to restore atrioventricular synchrony after systematic therapy. Using isoprenaline instead of a temporary pacemaker may lead to arrhythmia. Most FM patients with a conduction block recover after the acute stage. As the cardiac function could be worsen with right ventricle-only pacing in cardiogenic shock patient, the temporary pacemaker should be discontinued as soon as the normal atrioventricular conduction recovered. Eventhough the right ventricle-only pacing may contributed to a worsening hemodynamic status, we considered the deterioration of heart function is mainly caused by the cytokine storm in the myocardium of this patient. Permanent pacemaker implantation is considered when a conduction anomaly was still present after systemic treatment for 2 weeks. The respirator ensured adequate oxygenation during respiratory failure. CRRT can adsorb inflammatory molecules and toxins, correct the imbalance of electrolytes, acids and bases, and relieve the edema. If several days of mechanical circulatory support do not improve cardiac function, ventricular assist devices and heart transplantation should be actively considered.

A previous study showed that patients with FM had better outcomes than non-FM (6). Recently, this viewpoint has been challenged. A retrospective, international, multicenter cohort study published in 2019 including 220 patients with biopsy-proven myocarditis and left ventricular systolic dysfunction recognized that FM patients had higher rates of cardiac death and heart transplantation both in the short- and long-term, compared with non-FM patients (16). Julie et al. (20) indicated that a low pH, high lactate level, cardiac functional recovery time, malignant arrhythmia, high peak coronary markers, and immunoglobulin use were independent predictors of FM mortality. Our data showed that the combination of mechanical circulatory support and immunosuppression therapy reduced the mortality of FM from nearly 50% to 10%, compared with the conventional therapy (21). Interestingly, the patient's ventricular function fully recovered during follow-up.

In conclusion, FM is characterized by sudden onset, rapid progression, and unstable hemodynamics. The early initiation of mechanical circulatory support and immunosuppression are life-saving tools for its treatment. The outcome and prognosis of FM depend on the aetiology, clinical presentation, disease stage, and therapeutic strategy.

## Data Availability

The raw data supporting the conclusions of this article will be made available by the authors, without undue reservation.

## Supplementary material

SUPPLEMENTARY VIDEO 1

Transthoracic echocardiography showed (A) diffuse ventricular hypokinesia (LVEF 26%) and few pericardial effusion on admission, and (B) a normal cardiac chamber size and ventricular motion, with a LVEF of 58% on day 15 of hospitalization.
